# Effectiveness of the Green Heart Smartphone Application as a Self-Management Intervention for Hypertension and Dyslipidemia: A Randomized Clinical Trial

**DOI:** 10.34172/aim.28501

**Published:** 2024-05-14

**Authors:** Mojgan Ghavami, Alireza Abdshah, Saeed Sadeghian, Ayat Ahmadi, Mohammad Sajad Jolani, Diba Akbarzadeh, Fateme Haji Ali Asgari

**Affiliations:** ^1^Cardiovascular Research Institute, Tehran Heart Center, Tehran University of Medical Sciences, Tehran, Iran; ^2^Department of Public Health Sciences, Miller School of Medicine, University of Miami, Miami, FL, USA; ^3^School of Medicine, Tehran University of Medical Sciences, Tehran, Iran; ^4^Knowledge Utilization Research Center, Tehran University of Medical Sciences, Tehran, Iran; ^5^Student Research Committee, School of Medicine, Shahid Beheshti University of Medical Sciences, Tehran, Iran; ^6^Department of Information Technology, Virtual School, Tehran University of Medical Sciences, Tehran, Iran

**Keywords:** Dyslipidemia, Hypertension, Mobile-health, Secondary prevention, Smartphone, Technological interventions

## Abstract

**Background:**

Cardiovascular disease (CVD) is a major global health concern, the leading cause of death and disability. Thus, preventive interventions targeting modifiable risk factors are essential. Mobile-health technologies have emerged as promising tools for improving prevention by modifying risk factors. We created the "Green Heart" mobile app to help coronary artery disease (CAD) patients control their risk factors. The app has three modules: smoking cessation, dyslipidemia (DLP) control, and blood pressure (BP) management. This study evaluated the app’s performance in monitoring hypertension (HTN) and DLP among known CAD cases.

**Methods:**

A randomized controlled trial enrolled 1590 CAD subjects, including 1114 hypertensive patients and 1488 subjects with DLP, and assigned them randomly to paper-based education or application-based groups.

**Results:**

Regarding HTN, after 6 months, we finally analyzed 545 and 546 hypertensive patients, assigned to the conventional and app groups, respectively. Patients in the app group were more likely to have their BP managed successfully (88.6% vs. 78.5%; *P*<0.001). The app group showed higher odds of successful BP management (odds ratio [OR]: 2.13; 95% CI: 1.51 - 3.03). Regarding DLP, we analyzed 728 patients in the conventional and 714 patients in the app group. A higher percentage of patients in the app group (24.8%) had low-density lipoprotein cholesterol (LDL-C) levels less than 70 mg/dL (16.1%; *P*<0.001). The app group showed higher odds of reducing LDL-C (OR: 1.72; 95% CI: 1.32–2.26).

**Conclusion:**

We found that using the Green Heart app in the self-monitoring setting significantly improved BP and DLP management across the study population.

## Introduction

 Cardiovascular disease (CVD) is declining in developed countries, but rapidly growing in middle- and low-income countries.^1,2^ Studies in Europe and the United States show that 50%-60% of the drop in ischemic heart disease deaths is due to prevention strategies at the population and individual levels.^3,4^

 CVD is the leading cause of death and disability in Iran, accounting for nearly half of all deaths.^5^ Given that the Iranian population is young and will enter the high-risk age group for CVD in the coming decades, the death rate and disability from these diseases are projected to be severe. Another concern is that CVD affects the productive workforce under age 55 in Iran.^6-8^

 If the current trend in developing countries continues, their health systems will face a major challenge in managing the burden of CVD. The leading causes of CVD can be prevented by addressing associated risk factors such as high blood pressure (BP), high cholesterol, smoking, diabetes, obesity, and physical inactivity. Adopting healthy behaviors can prevent CVD deaths and illnesses.^9^ A recent analysis has revealed that a 5-mm Hg reduction of systolic blood pressure (SBP) in hypertensive patients leads to a reduction of 10% in cardiovascular attacks, 13% in stroke chance, and 5% in cardiovascular death.^10^

 A growing body of literature emphasizes the role of low-density lipoprotein-cholesterol (LDL-C) and apolipoprotein-B-containing lipoprotein in the pathogenesis of atherosclerosis. Atherosclerosis and its complications such as cardiac muscle infarction and ischemic stroke are known as the first agents of morbidity and mortality across the world.^11^

 Pharmacological and non-pharmacological approaches are widely used to control hypertension (HTN) and plasma lipid profile. Besides the preventive effects, lifestyle modifications have supplementary therapeutic effects. Regular daily aerobic physical activities, a healthy low-salt and vegetable-rich diet, eliminating saturated or trans fats, weight loss, and quitting smoking and alcohol consumption are well-investigated approaches for non-pharmacological treatment of HTN and dyslipidemia (DLP). By evaluating the patient’s situation, physicians choose one or more antihypertension and anti-lipid drugs to control the disease.^12,13^

 Therefore, effective preventive strategies with broad coverage, feasibility considerations, and applicability to different groups of people are needed to seriously address CVD risk factors. Traditionally, this task was carried out only by the healthcare system and physicians, which was costly and complex. Smartphone applications are gradually becoming useful assistants for chronic disease monitoring. These widely used and cost-effective applications remove the distance barrier to medical services. The simplicity and affordability of this technology encourage patients to continue monitoring their BP and DLP. Variations in these applications to accommodate the user’s language and ethnicity make them compatible with local users and improves the HTN and DLP follow-up process significantly.^14^

 Recent randomized controlled trials (RCTs) conducted in the Asian population including Iran have indicated that mobile-health (mHealth (app-based interventions hold promise for enhancing patients’ health knowledge and supporting self-management practices related to medication adherence, low-salt diets, and physical exercise, ultimately contributing to better BP control.^15-17^ They can also be used as an adjuvant method among patients with coronary heart disease for secondary prevention.^18^

 A recent systematic review has demonstrated that mobile-based health interventions designed to encourage physical activity and promote healthy lifestyle changes can lead to improvements in cardiometabolic risk indicators among individuals with metabolic syndrome. However, further research is necessary to facilitate a comparison between traditional clinical approaches and new interventions utilizing mHealth technologies. The limited availability of comparable RCTs may account for the observed results, as these technologies are relatively new. It would also be valuable to assess the individual effects of each component of these interventions and standardize the implementation of multicomponent interventions to generate sufficient evidence for inclusion in clinical practice guidelines.^19^

 Regarding the lack of these technologies in Iran, we designed the “Green Heart” smartphone application to assist coronary artery disease (CAD) patients with risk factor modification (HTN and DLP management and smoking cessation). This study aimed to evaluate the performance of the “Green Heart” application in HTN and DLP monitoring among known cases of CAD.

## Material and Methods

 We designed a parallel-group single-blinded RCT study to evaluate the impact of using a mHealth smartphone-basedapplication (app) on risk factor control in patients with CAD.

 From November 2022 to February 2023, we included individuals aged 25 to 75 years who attended Tehran Heart Center (THC) with documented CAD by coronary angiography and had at least one uncontrolled risk factor out of HTN, DLP, and current cigarette smoking (CS). THC is an academic tertiary-care hospital specializing in cardiovascular disorders in Iran affiliated with the Tehran University of Medical Sciences.^20^

 A smartphone-based application, named “Green Heart” was designed by our team in 2022 to help control cardiovascular risk factors for secondary prevention in CAD patients. The application consists of three modules to control DLP, manage BP, and quit smoking. In this study, we evaluate the impact of the two modules of HTN and DLP on risk factor control.

 The module of the app that targets HTN includes educational video clips to improve the knowledge of users, e.g. the risks and consequences of uncontrolled HTN and benefits of BP control, accurate technique of home BP measurement, guidance to choosing an appropriate sphygmomanometer and cuff size based on the size of the arm circumference and also advice about lifestyle modification such as low-salt, low-fat, low-sugar, and high-fiber diets, increasing the level of physical activity, weight loss, and quitting smoke. This module supports self-monitoring of BP. In this regard, picture guidance is provided for entering systolic and diastolic BP (DBP) based on the monitor screen of the digital devices. Patients using an input form with the possibility for refresh/ reinsertion of information, record their BP twice a day, seven days a week. The app keeps track of systolic and diastolic BP and displays trends of each user with graphs and charts over time. Thereafter, it averages one-week recorded BP and recommends visiting the doctor or taking medications at an appropriate time. Automatic feedback is provided on the entered values by showing alerts and color codes for specific ranges. Reminders are provided in the form of daily notifications at specific times to increase medication adherence.

 The module of the app which targets DLP includes educational content in the context of video clips about the risks of uncontrolled DLP and the benefits of lipid control and also information about healthy lifestyle and its impact on lipid levels reduction and heart disease risk elimination. This module supports self-monitoring of LDL-C. In this regard, a space for entering the value of LDL-C is designed. The user enters the value based on his/her lab test sheet according to the provided picture guide. Thereafter, questions about the type of lipid-lowering medication (statin/ ezetimibe, etc.) and its daily dose are asked, and if the entered value is higher than the target, a corresponding message is sent to him/her about the necessity of referring to the doctor for medication dose modification or another category of medication addition and if the entered value is optimal, it is recommended to continue the drug and perform a lab test again after 12 months. Daily pop-up notification messages remind medication intake at appropriate times to increase adherence.

 For developing the app, the American College of Cardiology/American Heart Association (ACC/AHA) Guideline^21^ on arterial HTN and European Society of Cardiology/ European Atherosclerosis Society (ESC/ EAS) Guidelines^22^ on the prevention of DLP were incorporated into the algorithm of the app. These guidelines were converted into computer-interpretable functions and applied to assessment and feedback systems.

 Connecting to the internet is not necessary during the process of entering data, viewing movies, and receiving advice and reminders. Whenever the user connects to the internet, the data is collected and saved to the server.

 The programming languages to design web service and data management panel and for mobile software were PHP 8.1 and Kotlin 1.7, respectively.

###  Sample Size Calculation

 Farkouh et al^23^ conducted a study that analyzed data from large randomized trials to assess the effectiveness of lifestyle modifications and medication use in controlling risk factors in patients with CAD for secondary prevention. To increase the success rate of modifying risk factors by at least 1.5 times, with a significance level of α = 0.05, a study power of 80%, and accounting for a 10% loss of samples, sample sizes of 257 for DLP, 538 for HTN, and 772 for CS were determined. The largest sample size, 772, was chosen for CS (resulting in a total of 1544 participants when doubled). The sample size calculations were conducted using the G-power software, and the final results are presented in Table 1.

**Table 1:** Success Rates in Controlling Dyslipidemia, Hypertension and Smoking and Calculated Sample Size

**Risk Factor**	**Success Rate Percentage in** **the Control Group**	**Success Rate Percentage in** **the Intervention Group**	**Sample Size in Each Group** **Counting 10% Shedding of Samples**	**The Abundance of Patients Referred** **to the Tehran Heart Center**
Dyslipidemia	0.2	0.3	257	0.72
Hypertension	0.11	0.165	538	0.47
Cigarette smoking	0.08	0.12	772	0.34

**Figure 1:**
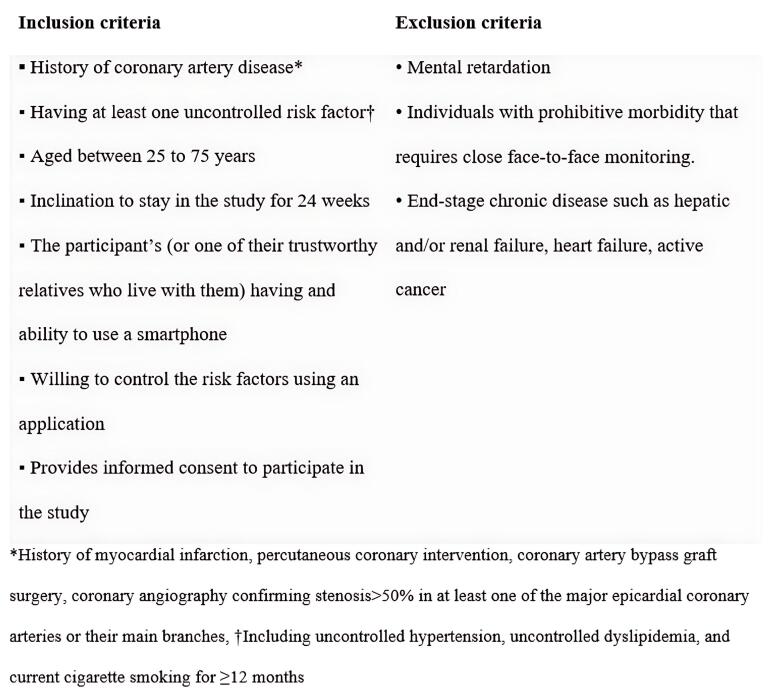
Eligibility Criteria for the Participants

 According to the eligibility criteria shown in Figure 1, we enrolled 1590 patients. To ensure a balanced representation of risk factors, participants were randomly assigned to study arms using a stratified block randomization method. Participants were categorized into seven distinct groups based on the presence and combination of key risk factors: (1) CS only, (2) HTN only, (3) DLP only, (4) CS and HTN, (5) CS and DLP, (6) DLP and HTN, (7) CS, HTN, and DLP. Within each group, randomization to the control arm (conventional paper-based treatment; 795 subjects) and intervention arm (Green Heart app; 795 subjects) was conducted with a 1:1 allocation using variable block sizes of 2, 4, 6, 8, and 10. A computer-generated random number list was used for permuted block stratified randomization (Block stratified randomization Windows Version 6.0, by Steven Piantadosi, M.D., Ph.D., Cedars-Sinai Medical Center). The practitioners who assessed the status of risk factor control were blinded to group assignment.

**Figure 2:**
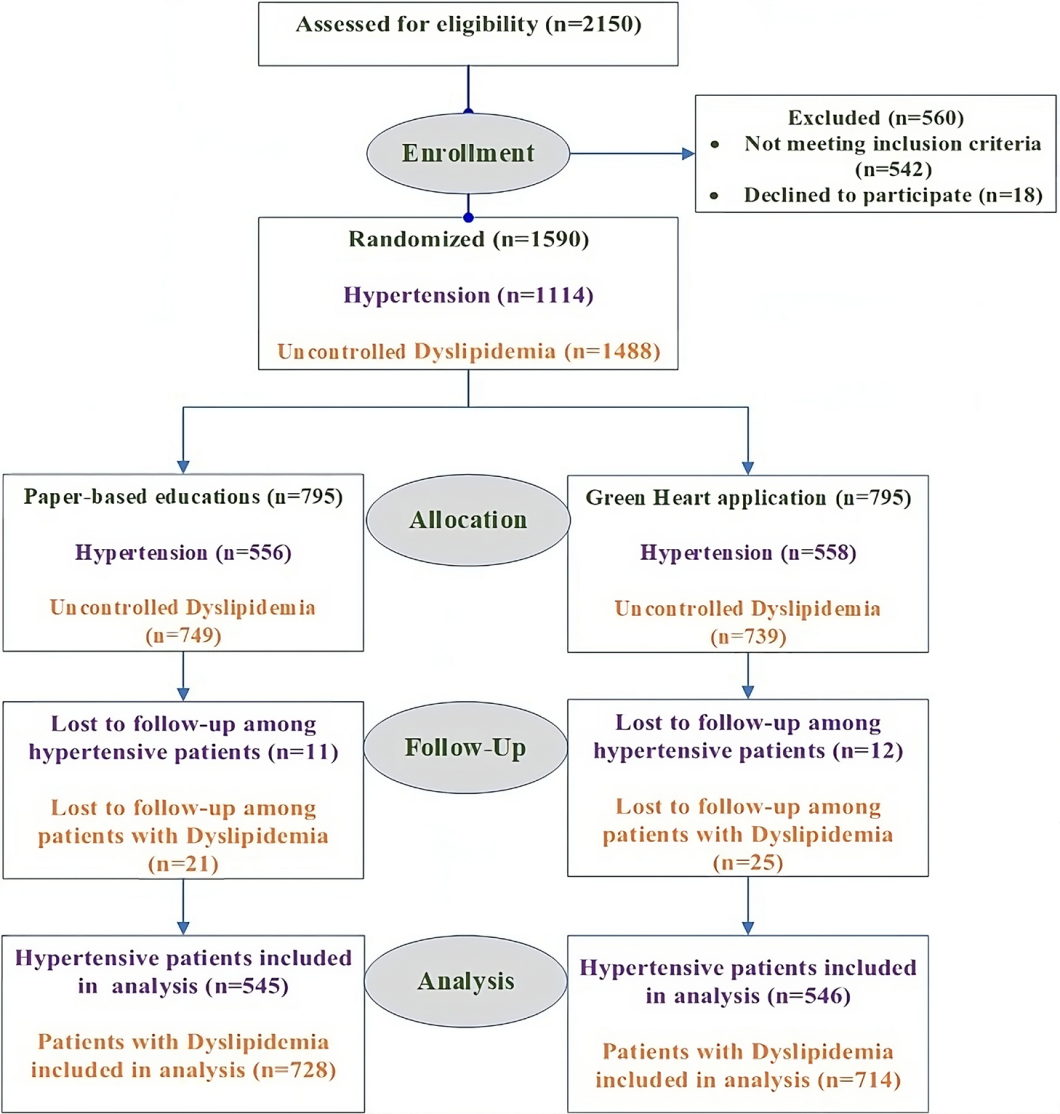
Consort Flow Diagram of Green Heart Trial

 Among the participants, 545 and 728 patients in the conventional group and 546 and 714 patients in the application group had HTN and DLP, respectively. Details are described in Figure 2. Patients in both groups received routine care (as described in the following).

###  Definitions

- Routine care: All the patients’ demographic and clinical characteristics (including past medical history, duration of each disease, drug and habitual history, etc) and the status of their risk factors (controlled/uncontrolled) were recorded in the baseline visit. BP was measured during the first visit and follow-up visits. The blood sample was taken before each visit to measure the serum levels of LDL-C. In addition, we requested all the patients to present every laboratory test report done even outside THC, and all other documents related to any event that required hospitalization between the visits.
- Hypertension: HTN classification is based on accurate BP measurements, with Stage 1 characterized by SBP ranging from 130 to 139 mm Hg or DBP between 80 and 89 mm Hg. Stage 2 is identified by SBP ≥ 140 mm Hg, or DBP ≥ 90 mm Hg.^21,24^
- Uncontrolled HTN: A history of HTN with/without using antihypertensive drugs is considered as HTN on the first visit. Uncontrolled HTN was defined according to the ACC/AHA guidelines as persistent BP ≥ 130/80.^21,24^
- Controlled HTN: Maintaining average systolic and diastolic BP records below 130 mm Hg and 80 mm Hg, respectively (treatment target^21^) at the time of office measurements and/or during one-week daily home measurements before clinic visits was considered as controlled. For office measurement, at the end of 6 months from randomization, an average of two BP readings on two separate occasions was obtained.
- Dyslipidemia: DLP is a term used to describe an imbalance of lipid levels including elevated total cholesterol, LDL-C, and triglyceride and/ or reduced level of high-density lipoprotein cholesterol. According to ESC/ EAS 2019, LDL-C is recommended for lipid analyses as a treatment target in the prevention of CVD. Elevated LDL-C levels threshold for treatment is defined based on individualized cardiovascular risk assessment. People with any of the following are considered as very high risk: Documented atherosclerotic CVD including previous acute coronary syndrome (myocardial infarction or unstable angina), stable angina and coronary revascularization (percutaneous coronary intervention, coronary artery bypass graft surgery, and other arterial revascularization procedures). So, the patients who were included in this study are considered as very high risk. For secondary prevention in very-high-risk patients, an LDL-C reduction of ≥ 50% from baseline and an LDL-C goal of < 55 mg/dL is recommended (Class of recommendation: I, level of evidence: A).^22^
- Uncontrolled DLP:Uncontrolled DLP description based on the serum level of LDL-C differs depending on the concomitant comorbidities including diabetes mellitus and CVD.^22^ The treatment target serum level of LDL-C is less than 55 mg/dL in significant CAD patients. Values more than the mentioned cut-off were considered uncontrolled DLP.
- Controlled DLP: Lowering serum level of LDL-C by < 55 mg/dL and ≥ 50% reduction from baseline^22^ in a follow-up visit at the end of 6 months from randomization is described as controlled. We also reported LDL-C levels less than 70 mg/dL according to the ACC/AHA guideline.^25^

At the baseline face-to-face visit, hypertensive participants in both arms were educated on the correct BP measurement method and all the participants received a healthy diet and physical activity plan. Hypertensive patients in the control arm received a paper-based chart to document their systolic and diastolic BP twice daily for 7 consecutive days in each month. In the intervention group, the software app was installed on the mobile devices of participants by our team. Participants and one of their trustworthy relatives who lived with them were also taught how to use the app. To resolve the problems, the patients were asked to work with “Green Heart” in the presence of our staff. To respond to any existing questions, we gave them a telephone number for technical support. During the study period, patients in the intervention group were encouraged to continue to use the app, enter and record the measurements, and follow recommendations for each risk factor in addition to routine care.

 The patients were followed for six months. Six months after randomization, the status of risk factors in both groups was evaluated in a follow-up visit. Hypertensive patients in the control group were also asked to bring their filled-out paper sheet BP chart of one-week daily home measurements for documentation.

 A cardiologist in the outpatient clinic documented any cardiovascular events, and modified medications. Any additional visits were considered for any patient requiring closer interval visits. In case of, an inability for participants to have a face-to-face visit, risk factor status was verified via a telephone call. The primary outcomes were defined as lipid control in patients with DLP and BP control in hypertensive patients. The secondary outcome was the number of participants who remained active users of the app till the end of 6 months.

 To measure the activity of the users, logging “system-usage” data and the number of filled-out self-report questionnaires were recorded.

 The success of each intervention on HTN management and lipid control was compared after 6 months. The data was analyzed using R studio and packages base R,^26^ tidyverse,^27^ and nnet.^28^

 We used the chi-square test (or Fisher’s exact test where appropriate) and t-test (or Kruskal-Wallis as a non-parametric equivalent where appropriate) to compare the distribution of categorical and continuous variables, respectively. We then conducted a multivariable analysis to adjust for baseline differences between subjects in age, sex, smoking, and diabetes mellitus. A *P* value of 0.05 was considered significant throughout this study.

## Results

###  Hypertension

 We finally analyzed the data of 1091 people with uncontrolled HTN at the start of the study. We assigned 545 to the conventional treatment group and 546 to the “Green Heart” application group. Table 2 summarizes the patients’ baseline characteristics (Section A: HTN) by group assignment.

**Table 2:** Baseline Characteristics of the Patients in the Hypertension study (Section A), and Dyslipidemia Study (Section B) Based on their Assigned Treatment

**Section A: Hypertension**		**Conventional Treatment(n=545)**	**Green Heart App(n=546)**	**Overall(N=1091)**	***P*** ** Value**
	Age				
	Mean (SD)	64.2 (7.71)	61.2 (8.40)	62.7 (8.20)	< 0.001
	Median [Min, Max]	65.0 [36.0, 75.0]	61.0 [38.0, 75.0]	63.0 [36.0, 75.0]	
	Sex*				
	Female	145 (26.6%)	114 (20.9%)	259 (23.7%)	0.031
	Male	400 (73.4%)	432 (79.1%)	832 (76.3%)	
	Uncontrolled dyslipidemia*	527 (96.7%)	528 (96.7%)	1055 (96.7%)	1
	Smoking*	212 (38.8%)	225 (41.2%)	437 (40.0%)	0.602
	Diabetes Mellitus*	252 (46.2%)	211 (38.6%)	463 (42.4%)	0.011
	Systolic blood pressure				
	Mean (SD)	142 (9.65)	139 (8.40)	141 (9.14)	< 0.001
	Median [Min, Max]	139 [130, 169]	135 [130, 165]	137 [130, 169]	
	Diastolic blood pressure				
	Mean (SD)	88.1 (9.22)	85.7 (7.87)	86.9 (8.65)	< 0.001
	Median [Min, Max]	84.0 [78.0, 109]	82.0 [74.0, 110]	83.0 [74.0, 110]	
**Section B: Dyslipidemia**		**Conventional Treatment(n=728)**	**Green Heart App(n=714)**	**Overall (N=1442)**	***P*** ** Value**
	Age				
	Mean (SD)	63.8 (7.82)	59.1 (9.47)	61.5 (8.98)	< 0.001
	Median [Min, Max]	64.0 [36.0, 75.0]	59.0 [28.0, 75.0]	62.0 [28.0, 75.0]	
	Sex*				
	Female	173 (23.8%)	132 (18.5%)	305 (21.2%)	0.021
	Male	555 (76.2%)	582 (81.5%)	1137 (78.8%)	
	Hypertension*	527 (72.4%)	528 (73.9%)	1055 (73.1%)	0.202
	Smoking*	304 (41.8%)	325 (45.5%)	629 (43.6%)	0.601
	Diabetes Mellitus*	295 (40.5%)	231 (32.4%)	526 (36.5%)	0.002
	LDL-C				
	Mean (SD)	93.8 (32.5)	91.9 (28.7)	92.9 (30.7)	0.3
	Median [Min, Max]	86.0 [55.0, 233]	85.0 [55.0, 233]	86.0 [55.0, 233]	

LDL-C, Low-density lipoprotein cholesterol. *Data are presented by number (Percentage).

 After 6 months, the mean SBP and DBP in the conventional treatment group were 122 mm Hg and 75.3 mm Hg, respectively. The mean SBP and DBP in the application group were 121 mm Hg and 77 mm Hg, respectively. The differences between the two groups were clinically significant (*P* = 0.002 and *P* < 0.001 for SBP and DBP, respectively).

 Patients in the application group were more likely to have their BP managed successfully than those in the conventional treatment group (88.6% vs. 78.5%). This difference was statistically significant (*P* < 0.001) (Table 3, Section A).

**Table 3:** Risk Factor Status after 6 Months (outcome) in the Hypertension Study (Section A), and Dyslipidemia Study (Section B) Based on their Assigned Treatment

**Section A: Hypertension**		**Conventional treatment (n=545)**	**Green Heart App (n=546)**	**Overall (N=1091)**	***P*** ** Value**
	Systolic blood pressure				
	Mean (SD)	122 (11.4)	121 (6.45)	122 (9.31)	0.002
	Median [Min, Max]	125 [100, 170]	120 [87.5, 172]	120 [87.5, 172]	
	Diastolic blood pressure				
	Mean (SD)	75.3 (8.13)	77.0 (6.02)	76.1 (7.20)	< 0.001
	Median [Min, Max]	75.0 [60.0, 105]	75.0 [50.0, 102]	75.0 [50.0, 105]	
	Successful blood pressure control*	428 (78.5%)	484 (88.6%)	912 (83.6%)	< 0.001
**Section B: Dyslipidemia**		**Conventional Treatment (n=728)**	**Green Heart App (n=714)**	**Overall (N=1442)**	***P*** ** Value**
	LDL-C				
	Mean (SD)	89.0 (24.7)	89.5 (28.7)	89.2 (26.7)	0.803
	Median [Min, Max]	81.0 [34.0, 240]	82.0 [33.0, 233]	82.0 [33.0, 240]	
	LDL-C < 70 mg/dL*	117 (16.1%)	177 (24.8%)	294 (20.4%)	< 0.001
	LDL-C < 55 mg/dL*	14 (1.9%)	26 (3.6%)	40 (2.8%)	0.071
	Successful lipid control^*#^	0 (0%)	10 (1.4%)	10 (0.7%)	< 0.001

LDL-C, Low-density lipoprotein cholesterol. *Data are presented by number (Percentage). ^#^Lowering serum level of LDL-C by < 55 mg/dL and ≥ 50% reduction from baseline is considered a successful lipid control.

 The crude odds ratio (OR) for successful BP management in those who were assigned to the application group compared to conventional treatment was 2.13 (95% confidence interval [CI]: 1.51–3.03). After adjusting for age, sex, smoking and diabetes, this OR was 1.71 (95% CI: 1.2–2.43).

###  Dyslipidemia

 We analyzed the data of 1442 people with uncontrolled DLP at the beginning of the trial. We assigned 728 to the conventional method group and 714 to the application group. A summary of the patients’ characteristics at the baseline and their treatment successes based on the assignment are listed in Table 2 (Section B) and Table 3 (Section B), respectively.

 The mean LDL-C level after treatment was not statistically different between the two groups (*P* = 0.803).

 Among the 728 patients in the conventional intervention group, 16.1% (117/728) had LDL-C less than 70 mg/dL and 1.9% (14/728) had LDL-C less than 55 mg/dL after 6 months. None of these patients achieved successful lipid management, based on the definition used in this study. Among the 714 people in the application group, 24.8 % (177/714) had reached LDL-C levels less than 70 mg/dL. LDL-C level less than 55 mg/dL was achieved in 3.6% of patients (26/714). Based on the definition of treatment success, only 1.4% (10/714) had successful management of DLP. The differences in treatment success between the two groups were statistically significant (Table 3, Section B).

 Due to the small number of treatment successes based on the recent definition of successful LDL-C control, we could not establish an accurate OR. However, the crude OR of reducing LDL-C levels to below 70 mg/dL in those who were assigned to the application group compared to conventional treatment was 1.72 (95% CI: 1.32–2.26). After adjusting for age, sex, smoking and diabetes, this OR was 1.94 (95% CI: 1.48 - 2.54).

 Among the patients who installed the “Green Heart” app, 24% (135/546) and 17% (124/714) were active users in the HTN and DLP modules, respectively, based on the definition.

## Discussion

 Previous studies show that even in developed countries, despite the advances in hypertensive patient findings and therapeutic methods, the disease control rate has plateaued in the past decades.^29^ BP measurement and interpretation errors could be responsible for this plateau. While standard office-based measurement of the BP is widely used to detect and control the disease, conditions like white-coat HTN or masked HTN challenge proper treatment. Home-based blood pressure monitoring (HBPM) is an effective substitute method in these conditions. HBPM is an economical, easily conducted method and is more tolerated by the patients.^30^ Investigations demonstrate an immense association between HBPM compared to office-based measurements with CVD risk.^31^ HBPM was found to be effective in reducing the treatment’s resistance by the patients and allows patients to gain better insight into HTN management.^32^

 The major limitations of HBPM are technical measurement errors and the presentation of the blood BP by the patients to the physicians. However, using new technology like telemonitoring and telemedicine facilitates patient-doctor communication in the HBPM method.^33^

 We found that using the “Green Heart” application in the HBPM setting significantly improved BP control across the study population. According to our results, using multimedia approaches and adding educational content to the HBPM method alters disease-controlling outcomes. Our notifications about on-time anti-hypertensive drug intake and educational items in the application about HTN control and management, accurate BP measurement technique, and advice about lifestyle modification seem to positively impact the consumer’s BP control. Using a graphical chart and automated feedback, the application significantly improves the outcome in BP management.

 Our result was in line with previous studies about the efficacy of mobile phone applications in HTN management. In a meta-analysis, four out of six RCT studies that measured the effects of telemedicine, telemonitoring, telecare, or the implementation of apps on systolic and diastolic BP management found statistically significant improvements.^34^

 In terms of HTN management, evidence advocates the use of HBPM rather than office-based BP management. The current trend of using smartphone applications makes them a promising approach for the HBPM method.^35^ Meanwhile, the role of additional support like educational content, advice on lifestyle modification, and telemedicine properties still needs to be investigated and may improve the long-term outcomes of the HBPM method.^36,37^ Diversity and quality of the educational content, HBPM protocols, and cooperation of the patients possibly alter the effects of additional support on HBPM methods. The controversy on the effect of additional support to the HBPM application should be evaluated further and calls for better supervision of developing health applications by the healthcare system. Our results suggest that providing relevant, apprehensible educational content, visualizing the BP trend, and providing automated feedback from measured BP promote the efficacy of the HBPM in six-month follow-up.

 Our findings about the efficacy of the “Green Heart” application in the management of high LDL-C were inconclusive. Although our result showed a statistically significant difference between the study groups and application users had better results in the final LDL-C reduction rate, the observed difference could not be interpreted as a clinically significant difference. A possible explanation for this controversy is the strict goal of the final LDL-C level. When comparison was made with the goal of LDL-C < 70 mg/dL, the between-group differences became more prominent; nevertheless, further evaluations with larger study populations and longer follow-up times are needed to find the exact magnitude of our application’s effect on DLP management.

 Mobile phone applications have not been widely used for DLP management and simultaneous HTN control. Few studies have evaluated the impact of smartphone applications in this area.

 Previous studies demonstrated that using a smartphone as a communication method for DLP follow-up could be beneficial.^14^ Mobile phone applications have not been widely used for DLP management and simultaneous HTN control. Few studies have evaluated the impact of smartphone applications in this area. Similar to our findings, these interventions were found to only have limited benefit in DLP follow-up.^38,39^

 One RCT in Sydney, Australia, from 2017 to 2021 randomized 390 patients into two groups. The intervention group received a game-based mobile app called MyHeartMate for 6 months to promote lifestyle changes in CAD patients. The mentioned app was highly acceptable to patients with CAD, but it did not improve risk factors or lifestyle behaviors other than triglyceride levels.^40^

 A review of 26 studies found that mHealth interventions for secondary CVD prevention in older adults can improve health behaviors and medication adherence, especially when they involve short message service texting. However, barriers to implementation remain, such as affordability, usability, privacy, and security concerns. Additionally, more long-term studies are needed to determine the most effective types of mHealth for older adults. Given the aging population, developing and implementing effective, widely accepted, cost-effective, and time-efficient mHealth interventions to improve CVD health in this vulnerable group is a top priority.^41^

 Another systematic review which included 27 studies found that compared to the usual care group, the mHealth group had increased adherence to medical therapy, the ability to reach BP targets and exercise goals, and reduced anxiety. They also had increased awareness of diet and exercise. However, there was no difference in smoking cessation, ability to meet LDL-C targets, or hospital readmission.^42^

 A meta-analysis of 20 studies found that mHealth technology has a positive effect on patients who have experienced a coronary event. mHealth can help patients improve their exercise capacity, physical activity, medication adherence, physical and mental quality of life, and reduce readmissions for all causes and cardiovascular causes.^43^

 Another systematic review of 23 RCTs with 34,915 participants found that mHealth interventions can be effective in improving adherence to CVD medication. However, the results were mixed, with six RCTs finding no significant effect. The mHealth interventions used in the studies included text messages, mobile phone applications, and voice calls, either as single interventions or combined.^44^

 Considering these facts, developing a mobile application with better content and greater intention to increase drug adherence for DLP management seems to be rational. Follow-up time, types of intervention with the mobile phone (text message, email, call, application), and quality of the educational content could explain the diversity in the magnitude of these interventions’ effects.

 Proper education on how to use the application, using a convenient smartphone application, and sampling both study arms from the same medical center were the strengths of our study. Our application works properly in offline conditions and patients were reminded of medication, easily documented the BP result, and had access to the educational content even in offline conditions. Targeting the BP and lipid control concurrently, as two major risk factors of CVD, is another considerable characteristic of our application. Although our results demonstrate the efficacy of our application in the short-term management of HTN, more investigation with a longer follow-up is needed to compare the long-term differences between the application and conventional method of HBPM method.

 As discussed above, the main limitation of our study was the inconclusive findings related to DLP management. Another limitation that makes our result less generalizable is that we could not evaluate patients’ adherence to the app and the view count of the videos was not measurable.

 We observed significant p-values for age, sex, and diabetes in our study. Upon further investigation, we found that while the age difference was statistically significant, it did not hold clinical significance as the three-year gap between groups was deemed negligible. Additionally, our analysis revealed that a majority of patients in both groups were male, with similar proportions in each group despite statistical testing results. This observation may be attributed to the higher representation of male patients in our center’s referrals. Furthermore, we conducted separate analyses for patients with pre-existing DLP and HTN after randomizing the overall study participants. As a result, there were variations in sample sizes between the two groups, leading to slight discrepancies in our analyses. Due to the study design and the minor discrepancies observed, we opted not to dwell extensively on these differences and instead employed analytical strategies to mitigate their impact.

 The smoking prevalence was the same for both study arms and all of the participants had a documented ischemic heart disease. All participants were advised to have regular physical activity and a healthy diet, but still, some differences in factors like body mass index, diet and physical activity levels, treatment regimen, and other comorbidities like renal failure or thyroid dysfunctions might have confounded our results. Although all participants were educated on BP measurement and recording the results, manual data entry for the BP measurement by the patients was another weakness of our study and may have caused some biases.

 For enhanced applicability, it is important to exercise caution when generalizing the results of this study, taking into account potential variations in demographic characteristics among participants, such as ethnicity, socioeconomic status, as well as the level of societal awareness and literacy.

## Conclusion

 We suggest that using a mobile application for self-monitoring can be beneficial for managing chronic diseases. Engaging the new technologies of telemedicine and online consultation in the application may improve its efficacy and facilitate patient-doctor communication and disease control rates.
